# Comparative Pharmacoeconomic Effectiveness of Interleukin-17 Inhibitors for the Treatment of Ankylosing Spondylitis

**DOI:** 10.1134/S1607672923700291

**Published:** 2023-10-13

**Authors:** T. V. Dubinina, I. Z. Gaidukova, N. A. Sableva, K. V. Sapozhnikov, V. D. Sokolova, D. G. Tolkacheva

**Affiliations:** 1 Nasonova Research Institute of Rheumatology, Moscow, Russia; 2 Mechnikov North-Western State Medical University, St. Petersburg, Russia; 3 St. Petersburg Clinical Rheumatology Hospital no. 25, St. Petersburg, Russia; 4 Russian Presidential Academy of National Economy and Public Administration, Moscow, Russia

**Keywords:** ankylosing spondylitis, genetically engineered biological agents, biologics, interleukin 17 inhibitor, network meta-analysis

## Abstract

The objective of this study was to compare the clinical efficacy and cost-effectiveness of IL-17 inhibitors (SEC, IXE, NTK) in the treatment of adult patients with ankylosing spondylitis (AS) in the healthcare system of the Russian Federation. Materials and methods. The study is a sub-analysis of a previously published systematic review and network meta-analysis of the comparative efficacy of biologics in adult patients with AS in the Russian Federation. NNT values were calculated for BASDAI 50 and ASAS 20/40 after 16 weeks of therapy for all studied drugs. CpR was estimated for each biologic after 16 weeks and one year of therapy. Additionally, we carried out an assessment of the financial burden of the most cost-effective strategies for the treatment of AS. The use of NTK is characterized by an average of no more than three patients needed to treat to achieve one ASAS 20/40 or BASDAI 50 response, while on IXE and SEC no more than 4–5 patients need to be treated, depending on the estimated effectiveness criterion. According to CpR estimate, NTK is the most cost-effective IL-17 inhibitor for the treatment of AS, both after 16 weeks and after one year of therapy. The obtained results make it possible to compare the effectiveness of IL-17 inhibitors from a clinical and economic points of view and can be used both in decision making on treatment strategies for individual patients and at the population level when deciding on the reimbursement of drugs.

## INTRODUCTION

Ankylosing spondylitis (AS) not only has an adverse impact on the patient and patient’s family but is also accompanied by significant economic losses on the part of the state, as it mainly affects people of working age. During the natural course of AS, this disease causes progressive worsening of the quality of life of patients, limitation of their daily activities, and a persistent and irreversible loss of functions of the musculoskeletal system, which ultimately leads them to disability [1]. The economic burden of the AS for 2019 amounted to 21.9 billion rubles per population or 395.5 thousand rubles per one patient. The main part of the costs (60%) in the structure of economic losses was formed by the lost gross domestic product due to a decrease in the working capacity of patients, and the indirect costs amounted to 17.2 billion rubles [2].

To maintain the quality of life and working capacity of AS patients, early diagnosis and timely adequate therapy are required. Currently, doctors have a wide range of drugs aimed not only at suppressing inflammation but also at inhibiting structural changes in the spine and peripheral joints. The use of genetically engineered biological agents (biologics) is one of the most effective ways to achieve the above goals. To date, to treat AS, two groups of biologics comparable in efficacy [3–5] are used: tumor necrosis factor-α inhibitors (iTNF-α) (adalimumab, golimumab, infliximab, certolizumab pegol, and etanercept) and interleukin (IL) 17 inhibitors (iIL-17): netakimab (NTK), secukinumab (SEC), and ixekizumab (IXE).

The first biologics recommended for the AS treatment were iTNF-α. Despite their successful use, in some cases they did not allow achieving the goals of treatment (remission or low activity) [6]. iIL-17 became an adequate alternative to iTNF-α both in the initial administration and in the case of the development of resistance to them. In view of the relatively recent appearance of iIL-17 in clinical practice, a comparative analysis of their clinical and cost-effectiveness seems relevant.

The aim of the study is to compare the clinical and cost-effectiveness of IL-17 inhibitors (secukinumab, ixekizumab, and netakimab) in the treatment of adult patients with ankylosing spondylitis within the healthcare system of the Russian Federation.

## MATERIALS AND METHODS

At the previous stages, a systematic review and network meta-analysis of publications devoted to the study of biologics for the treatment of AS, registered in Russia, were performed [7]. The present study is a subanalysis of the obtained data with a comparative assessment of the clinical efficacy of iIL-17.

The criterion that allows obtaining information on the price–quality ratio is the number needed to treat (NNT). This index reflects the number of patients that need to be treated to prevent one negative outcome or achieve one favorable outcome relative to the reference drug or placebo. In the cost–benefit analysis NNT uses the cost per response (CpR) index, which is the cost of treatment of one patient until achieving the clinical outcome.

To calculate NNT, it is necessary to evaluate the binary outcomes of AS, which include ASAS (Assessment of Spondyloarthritis International Society) 20/40 and BASDAI (Bath Ankylosing Spondylitis Disease Activity Index) 50 [8–10]:

• ASAS 20 is an improvement by at least 20% and at least by 1 point on a numeric rating scale (NRS) of 0 to 10 in at least three of the four ASAS domains: pain score and NRS patient disease activity score; assessment of functional disorders according to the BASFI index (Bath Ankylosing Spondylitis Functional Index); assessment of AS activity according to the BASDAI index; and average of 5 and 6 questions. There should be no deterioration in the remaining domain.

• ASAS 40 is an improvement by at least 40% and at least 2 points in NNR from 0 to 10 in at least three of the four ASAS domains with no deterioration in the remaining domain.

• BASDAI is an AS activity index. Within the framework of this article, the proportion of patients who achieved an improvement by at least 50% on this index was considered.

Since these outcomes are positive in their content, a lower NNT value indicates a higher efficacy of the drug [11, 12].

According to the recommendations of the European Medicines Agency (EMA), ASAS 20/40 treatment response criteria are included in clinical trials as the main indices of efficacy for most drugs used in AS treatment [13–16]. At the same time, to decide whether therapy should be continued, ASAS recommends using the BASDAI 50 as one of the main criteria for the iTNF-α effectiveness [17].

As noted above, in this study we used the results of a systematic review and network meta-analysis [7], which were prepared in accordance with the established practice of performing such studies [18]. A literature search for the systematic review was performed in the PubMed and Embase databases. The publications on randomized clinical trials (RCTs) of phases II and III, which evaluated the efficacy and safety of biologics registered in the Russian Federation for adult patients with AS, were included (search updated on September 29, 2020). The systematic review included 15 placebo-controlled RCTs and one RCT comparing two biologics.

Within the framework of the subanalysis, only the studies that studied the results of the use of drugs from the iIL-17 group (NTK, SEC, and IXE) were selected. They are registered in the Russian Federation in the following dosages:

• NTK: 120 mg as two 1-mL (60 mg) subcutaneous injections once a week at 0, 1, and 2 weeks and then one every 2 weeks.

• SEC: 150 mg subcutaneously as initial dose at weeks 0, 1, 2, and 3; this dose is then given monthly as a maintenance dose starting at week 4.

• IXE: 80 mg every 4 weeks.

**Fig. 1.**
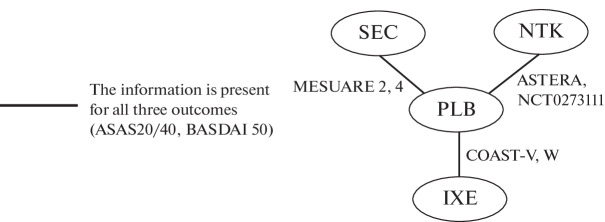
Subanalysis Evidence Network for IL-17 inhibitors. Designations: SEC, secukinumab; NTK, netakimab; PLB, placebo; IXE, ixekizumab.

The network of evidence (Fig. 1) in this case consisted of only indirect comparisons between placebo-anchored drugs (PLBs).

Evidence was synthesized using frequency network meta-analyses in RStudio v. 4.1.3 (RStudio, United States) in meta and netmeta packages. Before choosing the model parameters (fixed/random effects), heterogeneity and inconsistency were evaluated according to the I^2^ and Q criteria. Given the small number of degrees of freedom and the large scatter of heterogeneity values, we decided to use a network meta-analysis model with random effects. The effect of the drug was assessed compared to the PLB for the ASAS 20/40 and BASDAI 50 outcomes. To obtain the target index, NNT, the risk difference (RD) for the outcomes and its 95% confidence interval (CI) were used as the effect size. Because the ASAS 20/40 and BASDAI 50 outcomes are positive (i.e., targeted for the prescribed therapy), a positive RD value and its CI limits of a drug compared to PLB were considered as a positive result (benefit). If the RD (lower limit of its CI) was negative, a conclusion about a negative result (harm) was made.

NNT was calculated using formula (1):

1 $${\text{NNT}} = \frac{1}{{\left| {{\text{RD}}} \right|}};~\,\,{\text{RD}} = {{{\text{P}}}_{{{\text{TEST}}}}} - {{{\text{P}}}_{{{\text{CONTROL}}}}},$$

where NNT is the number needed to treat, RD is the risk difference of outcomes, P_TEST_ is the risk value in the main group, and P_CONTROL_ is the risk value in the control group.

The limits of NNT CI were calculated similarly on the basis of RD CI. The result was considered statistically significant if the NNT CI did not contain zero. The interpretation of NNT at positive values of both the central trend and both CI limits was identical to that of RD. At negative values of the CI limit, a conclusion was made about a “broken CI” of NNT and the departure of the CI limit from the area of the positive effect to the area of the negative one. CI was given with both positive limits and the corresponding “benefit”/“harm” marks. The features of CI in NNT were described in detail by Altman [19].

At the next stage, for all evaluated biologics, CpR for 16 weeks and 1 year of therapy was calculated based on the obtained NNT values. Although PLB in RCTs is used for a limited period of time and does not allow comparison of clinical efficacy of drugs and calculate NNT over a longer period of time, for the economic assessment of the cost of one year of treatment it was assumed that short-term responses to therapy would persist until the end of the year.

Data on maximum selling prices were obtained from the State Register of Maximum Selling Prices as of June 1, 2022, including 10% VAT [20]. The calculations also took into account the number of injections and the cost of drug therapy for 16 weeks and 1 year of induction therapy in accordance with Russian instructions for medical use.

To achieve the goal of the study, we analyzed the impact on the budget on a model population, which took into account CpR data for the annual course of induction therapy for each biological. At the next stage, we calculated the costs per population separately for each drug. Savings were calculated by evaluating the difference in costs between the biologicals. Then, we determined the number of additional patients that could be treated with the saved budget.

## RESULTS AND DISCUSSION

**Fig. 2.**
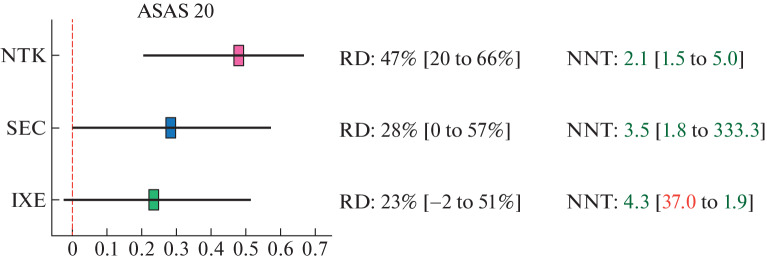
Result of the comparative effectiveness assessment of netakimab (NTK), secukinumab (SEC), and ixekizumab (IXE) for ASAS 20: negative lower limits of confidence intervals for NNT are marked in red (harm!) and imply a gap in interval NNT values.

**Fig. 3.**
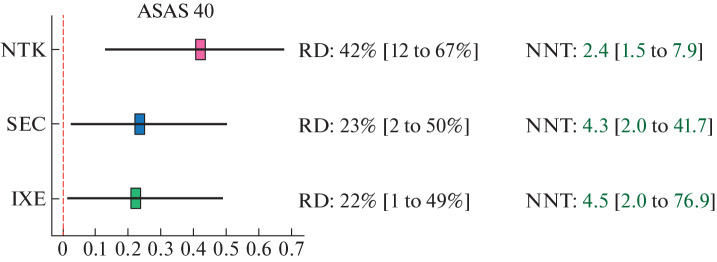
Result of the comparative evaluation of the effectiveness of netakimab (NTK), secukinumab (SEC), and ixekizumab (IXE) for ASAS 40.

**Fig. 4.**
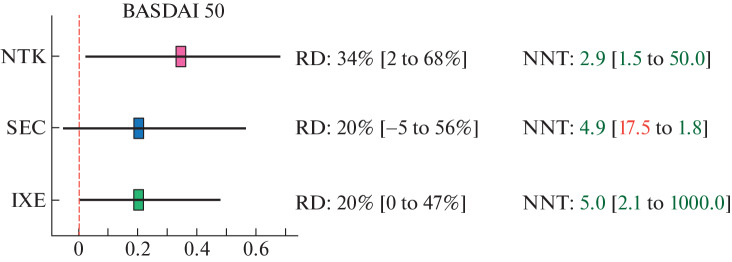
Result of comparative assessment of the effectiveness of netakimab (NTK), secukinumab (SEC), and ixekizumab (IXE) for BASDAI 50: negative lower limits of the confidence intervals for NNT are marked in red (harm!) and imply a gap in interval NNT values.

By the effect pooling results, all iIL-17 showed comparable results in terms of response rates to treatment (ASAS 20/40 and BASDAI 50) (Figs. 2–4). To obtain one response to therapy according to all three criteria (ASAS 20/40 and BASDAI 50), on average no more than three patients should be treated with NTK and no more than four or five patients should be treated with IXE. To obtain one ASAS 20 response to SEC, no more than four patients should be treated, and to obtain one ASAS 40 and BASDAI 50 response to SEC, no more than five patients.

Although a difference of 1 or 2 NNT units may seem insignificant, a difference of ±0.5 NNT units is considered clinically significant [21–24], especially taking into account the cost of one drug pack, which is quite high for biologics.

The next step was the calculation of CpR for 16 weeks and 1 year of therapy. Among the analyzed drugs, the average cost of obtaining one response was the lowest for NTK. For example, it was found that, to obtain an ASAS 20 and ASAS 40 response to SEC after 16 weeks of treatment, over 2 times more costs are required than for the response for NTK. IXE treatment was also 1.5–2 times more expensive. The cost of obtaining a BASDAI 50 response to NTK was less than that for IXE and SEC. The results are presented in Table 1.

**Table 1.** CpR evaluation for IL-17 inhibitors

Responses	Duration of therapy	Drug	CpR, thous. rubles
ASAS 20	16 weeks	NTK	400
		IXE	900
		SEC	900
	1 year	NTK	1100
		IXE	2900
		SEC	2100
ASAS 40	16 weeks	NTK	500
		IXE	900
		SEC	1100
	1 year	NTK	1300
		IXE	3000
		SEC	2600
BASDAI 50	16 weeks	NTK	600
		IXE	1000
		SEC	1300
	1 year	NTK	1600
		IXE	3400
		SEC	3000

CpR, cost per response; ASAS, Assessment of Spondyloarthritis International Society; BASDAI, Bath Ankylosing Spondylitis Disease Activity Index; NTK, nontokimab; IXE, ixekizumab; SEC, secukinumab.

The average Cost per Response (CpR) index is used to compare the cost-effectiveness of different drugs. Due to the difference in the costs of obtaining a response, using one of the drugs it is possible to treat a larger number of patients without changing the total budget for therapy. As an example, we estimated the number of patients who can be treated for the budget allocated for the treatment of 100 patients with the drug with the highest CpR. The result of this assessment for iIL-17 in terms of the number of patients who can be treated additionally (over 100) is shown in Fig. 8.

**Fig. 5.**
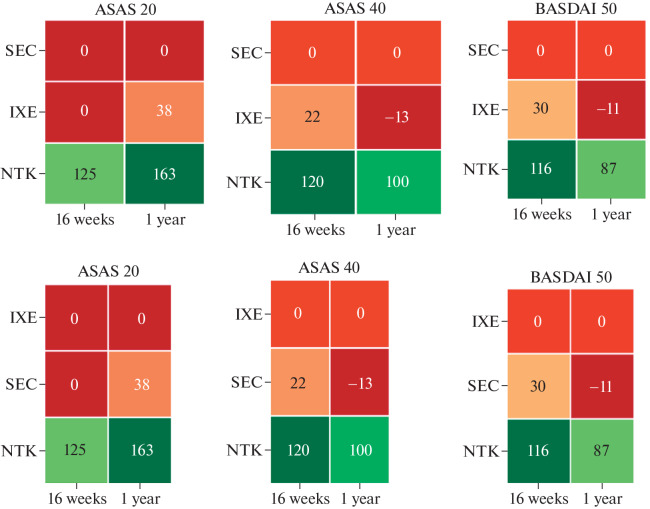
Number of patients additionally treated with IL-17 inhibitors per 100 patients: IXE, ixekizumab; SEC, secukinumab; NTK, nontackimab.

In the case of a fixed budget, if NTK is chosen to obtain an ASAS 20/40 response, approximately 100 patients can be treated in 1 year for the savings compared to the cost of SEC. Due to the difference in price between these drugs, obtaining an ASAS 20/40 response within 16 weeks for the same funds will also be available to more than 100 patients. Similarly, the cost-effectiveness of NTK exceeds that of IXE: for both 16 weeks and 1 year of therapy for an ASAS 20/40 response, the budget savings would allow an additional 100 patients to be treated. The benefit from the use of NTK to achieve BASDAI 50 for 16 weeks and 1 year of therapy is 116 and 87 patients, respectively, compared with SEC and 86 and 98 patients, respectively, compared with IXE.

IL-17 inhibitors occupy one of the leading positions among the drugs used for the treatment of immunoinflammatory diseases. However, no direct comparative RCTs between different iIL-17 have been performed. All available data on iIL-17 comparative efficacy are based on indirect comparisons in network meta-analyses [25–27], which implies a number of assumptions. Our knowledge about the effectiveness of iIL-17 and the short period of their use in clinical practice are limited. For example, SEC and NTK were registered in 2016 and 2019, respectively. Our results showed that all compared drugs had equal clinical efficacy; however, NTK showed the best combination of price and clinical efficacy among iIL-17 registered in the Russian Federation.

The data obtained do not contradict the results of a comparative assessment of the clinical efficacy of NTK in other immunoinflammatory diseases [26–28] and can be correlated with the previously published results of RCTs [29, 30] and Markov modeling [31] of the economic effect of NTK compared with other drugs. In a recent systematic review [26], NTK was shown to be highly effective in achieving target clinical outcomes in patients with psoriasis compared to PLB, which places it on a par with IXE and SEC. In the study of by Tolkacheva et al. [27], performed in 2021, NTK was shown to be no less effective than other iIL-17 in the treatment of psoriatic arthritis.

The absence of statistically significant differences in SEC to achieve BASDAI 50 and IXE to achieve ASAS 20 responses is due to the limitations of the present study shown below and does not mean the actual absence of an effect. Conversely, their effectiveness is supported by real clinical practice. It should also be taken into account the limited size of the accumulated evidence base for the effectiveness of NTK in comparison with the anchor (PLB) and the absence of direct comparisons of it with other drugs. At the same time, the full cycle of NTK production in the Russian Federation, as well as its high clinical and economic efficiency, open up certain prospects for its use in the treatment of adult AS patients.

The limitations of this study include the fact that the current NNT analysis is based on the published data of RCTs. Therefore, the patient population may not fully reflect the profile of patients observed in real clinical practice. In particular, all included studies had a mixed population of patients, both treated and not treated previously with biologics (except the COAST W study, in which the population was represented only by patients who received biological previously).

Despite the global recognition of network meta-analysis as one of the main tools of evidence-based medicine, this statistical method is based on indirect comparisons, which means that it is more sensitive to various distortions of RCTs included in it. This fact is confirmed by the results of the sensitivity analysis. At the same time, the used network meta-analysis [7] is the most correct of the available comparative studies of the effectiveness of biologics in patients with active AS in the Russian Federation.

Treatment of patients with active AS with only PLB is unethical [32, 33]. For this reason, in RCTs, in addition to the studied drugs and PLB, non-steroidal antiinflammatory drugs, basic antiinflammatory drugs (such as sulfasalazine or methotrexate), and glucocorticoids were used, which determined the need for us to use a model with random effects in meta-analysis.

Despite the significant improvement in the quality of life of AS patients treated with biologics [25], the assessment of the dynamics of this index during therapy was not included in the current analysis. We used the commonly accepted ASAS 20/40 and BASDAI 50 treatment outcome measures, recognizing that binary outcomes are the basis for calculating risk difference, NNT, and CpR.

Finally, when determining the cost-effectiveness, we did not take into account the direct medical costs associated with hospitalization and doctor visits, since all drugs are administered subcutaneously and in most cases do not require inpatient treatment. We also did not include in the analysis the cost of managing adverse events, due to the fact that, according to the results of a network meta-analysis [7], all the considered biologics had a comparable safety profile, and data on the comparison of the safety of all evaluated biologics during the year of therapy are missing. In our work, the survival of therapy was not taken into account, since all the studied drugs are used relatively recently, and data on their tolerability are currently being accumulated [25].

## CONCLUSIONS

In this study, it was found that all iIL-17 have a comparable high clinical efficacy in the treatment of AS, with NTK being distinguished by the lowest average cost per response (CpR). The results obtained can be used both in determining the treatment tactics for individual patients and at the population level when deciding whether a drug should be included in the reimbursement system and subsequent purchase.
